# A Simple Paper-Based Colorimetric Device for Rapid Mercury(II) Assay

**DOI:** 10.1038/srep31948

**Published:** 2016-08-24

**Authors:** Weiwei Chen, Xueen Fang, Hua Li, Hongmei Cao, Jilie Kong

**Affiliations:** 1 Department of Chemistry and Institutes of Biomedical Sciences, Fudan University, Shanghai 200433, P.R. China

## Abstract

Contamination of the environment by mercury(II) ions (Hg^2+^) poses a serious threat to human health and ecosystems. Up to now, many reported Hg^2+^ sensors require complex procedures, long measurement times and sophisticated instrumentation. We have developed a simple, rapid, low cost and naked-eye quantitative method for Hg^2+^ environmental analysis using a paper-based colorimetric device (PCD). The sample solution to which platinum nanoparticles (PtNPs) have been added is dispensed to the detection zone on the PCD, where the 3,3,5,5-tetramethylbenzidine (TMB) substrate has been pre-loaded. The PtNPs effect a rapid oxidization of TMB, inducing blue colorization on the PCD. However, Hg^2+^ in the solution rapidly interact with the PtNPs, suppressing the oxidation capacity and hence causing a decrease in blue intensity, which can be observed directly by the naked eye. Moreover, Hg^2+^ at concentrations as low as 0.01 uM, can be successfully monitored using a fiber optic device, which gives a digital readout proportional to the intensity of the blue color change. This paper-based colorimetric device (PCD) shows great potential for field measurement of Hg^2+^.

As a novel bioanalytical tool in recent years, paper-based analytical devices are widely used in environmental studies^1^,^2^, food safety analysis^3^,^4^,^5^ and molecular diagnostics^6^,^7^,^8^. Paper-based devices have many advantages compared with conventional sensors, including ease-of-use, cost-effectiveness, simple fabrication and biocompatibility^9^,^10^,^11^,^12^. As paper-based analytical devices represent a relatively new analytical platform, many effective strategies have been established for multiplex analyte measurement, such as electrochemical^13^,^14^ and chemiluminescent methods^15^, colorimetric methods^16^,^17^, FRET-based fluorescence methods^18^,^19^, and surface-enhanced Raman spectroscopy^20^,^21^. Among these strategies, colorimetric methods are well established because they are simple, quick to perform, and inexpensive. Various paper-based colorimetric device (PCDs) have been successfully developed in recent years. For example, Phillips *et al.*^22^ described a paper-based microfluidic devices platform with naked eye detection, which required no complex or expensive instrumentation. Whiteside *et al.*^23^ reported a paper-based ELISA platform with colorimetric detection that used a 96-microzone paper plate for the assay. In addition, there are many works focused on the use of smartphones as a new technology for the paper-based colorimetry^24^,^25^,^26^. By using smartphones, the colorimetric data can be rapidly converted to a digital image or a discrete number and provide an excellent solution for point-of-care (POC) test and real-time analysis.

One significant application in nanomaterials concerns the potential of enzyme mimetics^27^. Natural enzymes have many intrinsic drawbacks such as weak stability, high cost, and that they are easily influenced by environmental conditions^28^. So, naturally, some nanoparticles (NPs) with peroxidase-like or oxidase-like activities, such as graphene oxide (GO)^29^, single wall carbon nanotubes (SWNTs)^30^, carbon nanodots^31^, MoS_2_ nanosheets^32^, Fe_3_O_4_ magnetic NPs^33^, cerium oxide nanoparticles^34^, platinum nanoparticles (PtNPs)^35^ and gold nanoparticles (AuNPs)^36^, have been widely used as artificial enzymes for sensitive bioassay for their superior bioanalysis capacities over the past decade. Therefore, we have chosen PtNPs as a powerful nanozyme to catalyze the oxidation of TMB to generate signal amplification in colorimetric detection because of its excellent catalytic activity, good biocompatibility, easy synthesis and stability^37^.

As dangerous environmental pollutants, mercury(II) ions (Hg^2+^) are an ongoing environmental concern. In recent years, compact and portable instrumentation has become available for real-time and on-site trace Hg^2+^ screening of environmental and biological samples^38^,^39^,^40^,^41^,^42^,^43^. A novel lab-on-a-phone device with light-emitting diodes and dual wavelength illumination at 523 and 625 nm featured novel reaction chemistry based on Hg^2+^-induced aptamer probe conformation changing and citrate-stabilized gold nanoparticles (AuNPs) aggregation^44^. Paresh *et al.*^45^ reported a miniaturized and battery-operated device used to assay Hg^2+^ in water and in fish. The measurement principle was based on Hg(II) competing for binding with rhodamine B, whose fluorescence was quenched by AuNPs-based nanomaterial surface energy transfer (NSET). Meanwhile, as distinct from most schemes for Hg^2+^ analysis, application of colorimetric approach for a single particle-based sensing of Hg^2+^ have been developed recently which exhibited more attractive sensitivity^46^.

Recently, many works have discovered that mercury ions could interact with other metal nanoparticles to form bimetallic NPs^47^,^48^. In addition, it was also confirmed that the citrate around the surface of metal NPs was essential to promote the process of these combinations. In this work, we have introduced a novel design for a PCD that incorporated a Hg^2+^-induced color change and offered simple and real-time colorimetric detection of Hg^2+^. Our basic strategy of this PCD depended on the specific interaction between PtNPs and mercury ions and the Hg-Pt alloy can efficiently weaken its peroxidase-like catalytic activity. First, as showed in Fig. 1, the peroxidase-like activity of PtNPs is rapidly (2 minutes) inhibited through mixing and incubation with Hg^2+^ solutions. As a result of the oxidation of TMB, which yields a blue color, the presence of Hg^2+^ dramatically decreases the color intensity, which can be observed directly by the naked eye. Moreover, besides visual observation of the quantitative color change on the paper chip in response to the presence of Hg^2+^, a fiber optic laser device was also used to measure small changes in Hg^2+^ concentration. The fiber optic emits at a red wavelength, which is well absorbed by the blue-colored complex and the decrease in the intensity of reflected light is recorded by the electrical readout system. In this way, the high intensity of the blue color, which develops on the paper-chip, corresponds to a low digital readout representing the assay of Hg^2+^. This novel measurement approach provides a rapid and robust sensor technology and has considerable potential for trace Hg^2+^ analysis in diverse analytical scenarios.

## Results and Discussion

### Device design and colorimetric detection of Hg^2+^

In this work, we designed a novel PCD which contained three components as shown in schematic 1: (1) the circular patterned paper can be treated as a miniature reactor for PtNPs catalyzing the oxidation of TMB^49^; (2) a plastic framework with central hole was used for positioning the paper chip; (3) a fiber optic analysis system was able to read-out the fading of the blue colored signal response as a result of the alteration in peroxidase catalytic activity because of reaction of the PtNPs with Hg^2+^. In the context of rapid and on-site analysis of real samples, the TMB/H_2_O_2_ substrate solution was first dispensed on the paper-chip deposition zone. Then a small, widely-available plastic dropper bottle containing the sample solution with Hg^2+^ and PtNPs was used to initiate the reaction. A few minutes later, one drop of the mixture was added to the detection zone where the PtNPs oxidized the TMB to induce the formation of a blue-colored sediment on the paper-chip. However, as the Hg^2+^ presenting at water sample and the peroxidase-like activity of PtNPs would be rapidly inhibited after mixing and incubating with the solution, thus the blue oxidation of TMB decrease remarkably that could be viewed directly by the naked eye. The intensity of the blue color change for the PCD could readily be registered by digital camera or smart phones and the fiber optic system was used for signal quantitation by calculating the amount of light reflected from the detection zone by the paper surface. Because of key features such as simplicity of operation, convenience and rapid signal response, we believed this approach offers great potential for real-time, determination of Hg^2+^ ions in diverse applications^50^.

### Paper-based colorimetric assay

**Figure 2:**
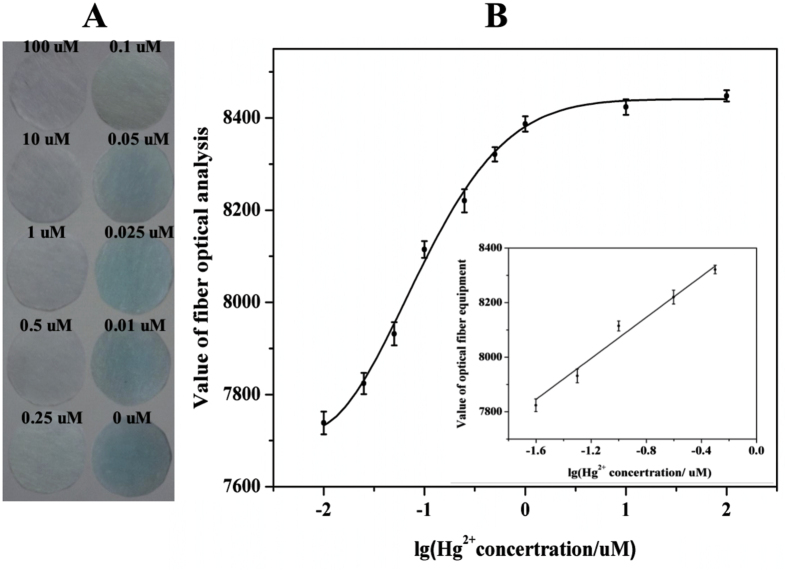
Colorimetric results for Hg^2+^ determination. (**A**) Images for the PCD displayed the peroxidase-like inhibition of different Hg^2+^ amounts. (**B**) The calibration curve indicated the correlation between value of optical fiber reader and the logarithm of Hg^2+^ concentrations.

For accurate and sensitive analysis, solutions with known concentrations of Hg^2+^ ions were added to the storage-bottle where PtNPs had already been diluted to adequate volume. Following gentle shaking for 2 min, one drop was immediately dispensed onto the detection zone of the PCD which already had 10 μL of TMB/H_2_O_2_ solutions loaded. The detection zone was visually evaluated within 5 min to ensure oxidation of TMB. This simple operating procedure and short measurement time fulfilled key requirements for rapid, portable and field measurement of Hg^2+^. Samples with higher Hg^2+^ concentrations gave higher intensity readings in the fiber optic analysis equipment. In Fig. 2B, the values increased as the target concentrations increased then values gradually reached steady-state at analyses concentration of ca. 0.5 μM, which corresponded, by visual inspection, to images of patterned papers of low color intensity. Comparing the correlation between color change and the amount of Hg^2+^, the color of the paper-chip changed from dark blue to light blue when the concentration of Ha^g+^ increased. Furthermore, it is worth noting that the colors of the patterned papers were almost colorless until the target concentration was below 0.1 uM, which demonstrated that Hg^2+^ ions can strongly inhibit the peroxidase-like activity of PtNPs. In addition, it also can be observed in the inset of Fig. 2B that the value of optical fiber reader exhibits a good linear correlation with the logarithm of Hg^2+^ concentration over the range from 0.025 um to 0.5 um. The correlation equation was V = 8446.772 + 375.773 lg C, where V was the response value of the fiber optical device and C was the concentration of Hg^2+^. The results demonstrated that this PCD could accurately discriminate Hg^2+^ concentration at 0.01 uM, which make it suitable for monitoring low content Hg^2+^ in water samples. The reproducibility and stability of PCD was good since similar results were achieved for the three replicate analyses.

### Selectivity of PCD for Hg^2+^ Determination

**Figure 3:**
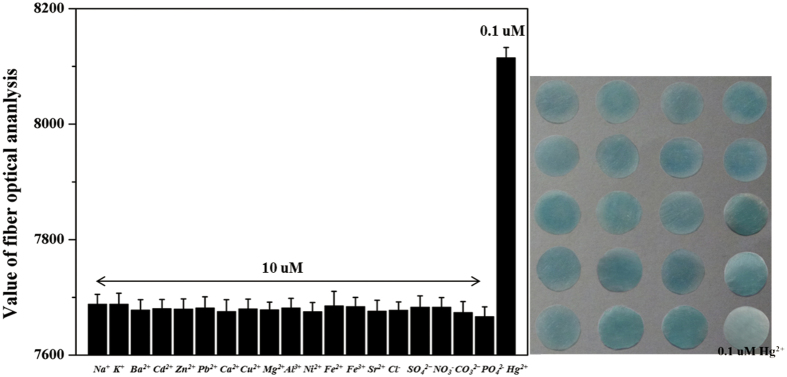
Selectivity of PCD for detection of Hg^2+^ (0.1 uM). Concentrations of metal ions and other anions, 10 μM. The photograph shows PCD colorimetric response to various control ions and Hg^2+^ (indicated by the bold type words).

The specificity of the method was investigated by viewing the response to other environmentally relevant metallic ions (Na^+^, K^+^, Ba^2+^, Cd^2+^, Zn^2+^, Pb^2+^, Ca^2+^, Cu^2+^, Mg^2+^, Al^3+^, Ni^2+^, Fe^3+^, Fe^2+^, Sr^2+^) and other common anions (Cl^−^. SO_4_^2−^, NO_3_^−^, CO_3_^2−^, PO_4_^2−^) under the same experimental conditions as used for Hg^2+^ sensing. The final concentration of Hg^2+^ ions and other ions was adjusted to 0.1 uM and 10 μM, respectively. By comparing the color intensities of the paper chips for different metallic ions and some anions, we found that all controlled ions did not exhibit inhibitory effects on the PtNPs, with the result that large blue precipitates formed within several min. as a result of catalysis of the TMB-H_2_O_2_ reaction. Also, as shown in Fig. 3, the metal ions gave appropriate response signals when the fiber optic module was used. Clearly the presence of Hg^2+^ in these samples would induce a large increase in readout value. These results indicated that the approach offers excellent selectivity for Hg^2+^ detection and is well suited to field monitoring of environmental waters. However, probably due to lacking enough sensitivity, our PCD platform showed less positive response to S^2−^ ions. We found that S^2−^ ions performed slight inhibitory activity to PtNPs only its concentration was less than 0.1 uM (This dates were not listed on the Fig. 3).

### Detection of Hg^2+^ In Real Samples

**Figure 4:**
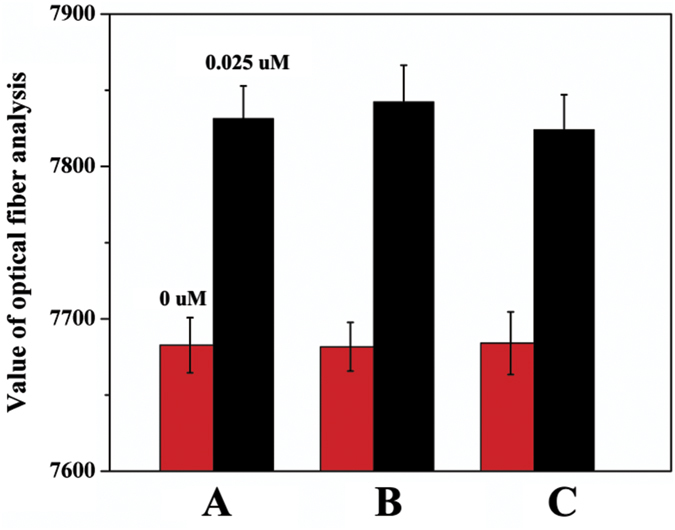
Real samples colorimetric assay by PCD with 0.025 uM Hg^2+^ (black) and no Hg^2+^ (red). (**A**) standard solution for Hg^2+^ analysis. (**B**,**C**) Hg^2+^ spiked pond and tap water samples analysis.

On the basis of the sensitivity and selectivity studies, the practicality of deploying the PCD device for real water sensing was investigated. For this purpose, the signal intensities of Hg^2+^ spiked pond and tap water samples were compared with those achieved for standard solutions of Hg^2+^ (0 uM or 0.025 uM), analyses being performed in triplicate. Figure 4 shows that the response values from pond and tap water samples were in good accord with the results of the standard solutions. Recovery ranged from 88% to 98.4% (Table S1) were analyzed by the addition and recovery experiments, which indicated that this new sensing platform would be suitable for the assay of trace Hg^2+^ in real samples.

## Conclusions

In summary, we have developed a portable PCD that utilizes PtNPs as a signal amplification probe for enzymatic oxidation of TMB whereby a colored product is produced on sensing paper. The device is also combined with a fiber optic module to give a digital readout of color intensity. The PCD permits determination of Hg^2+^ at concentrations as low as 0.01 uM within several minutes. Additionally, the approach does not depend on expensive instrumentation and the simple operating procedures mean that non-specialists can perform the analysis. Given that this paper-based platform offers advantages of low-cost, convenience and near real-time analysis^51^, the approach has great potential as a new tool for field measurement of Hg^2+^ particularly in underdeveloped countries.

## Methods

### Materials and reagents

Trisodium citrate (Na_3_C_6_H_5_O_7_ · 2H_2_O) and 3,3′,5,5′-tetramethylbenzidine (TMB) were purchased from Sigma-Aldrich. (St. Louis, MO). Hydrogen peroxide (30% w/w H_2_O_2_) and H_2_PtCl_6_ · (H_2_O)_6_ were obtained from Sinopharm Chemical Reagent Co. Ltd. (Shanghai, China). Glass fiber paper was purchased from Shanghai Kinbio Tech. Co. Ltd. Other reagents and chemicals were at least of analytical reagent grade and used without further purification. The TMB/H_2_O_2_ colorimetric substrate solution consisted of 2% v/v TMB (30 mM) and H_2_O_2_ (30% w/w), 140 mM NaAc in 20 mM Tris-HAc buffer (pH 4.0). All stock solutions were initially prepared by dissolving reagents in high purity water (Millipore water, 18.3 MΩ).

### Synthesis of citrate-capped PtNPs

The synthesis of citrate-PtNPs was based on a previously reported procedure that used a citrate reduction method^37^. First, all glassware and a magnetic stirrer bar were cleaned in aqua regia (HCl/HNO_3_ 3:1 v/v), then rinsed with ultrapure water several times and oven-dried overnight. 7.78 mg of platinum chloride (H_2_PtCl_6_ · 6H_2_O), dissolved in deionized water (40 mL), was vigorously mixed with trisodium citrate (0.4 mL, 100 mM). After stirring for 30 min at room temperature, freshly prepared aqueous sodium borohydride (200 μL, 50 mM) was added dropwise to the mixture. Next, the mixture was stirred slowly until the color of the solution remained stable. Finally, the colloidal solution was stored at 4 °C in the dark. The stored solution of PtNPs was still color-uniformity, almost no aggregations and precipitates can be found during half a year period.

### Preparation and design of PCD

**Figure 5:**
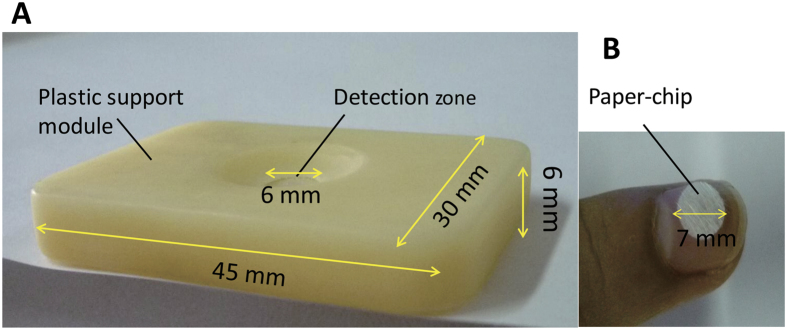
Components of the portable paper-based colorimetric device (PCD). (**A**) Stereo structure of rectangular plastic module (45 × 30 × 6 mm^3^) containing a round-hole (6 mm diameter). (**B**) Image of Hydrophilic glass fiber paper-chip with a diameter of 7 mm.

The PCD design and features are displayed in Fig. 5, a previous report describing the printing technique used for device fabrication^19^. A circular-shaped paper chip of 7 mm diameter was designed using a drawing software package that was connected to a cutting plotter (Silhouette GAMEO, made in Vietnam). Subsequently, we designed a rectangular plastic module with a round-hole (6 mm diameter) as a detection zone which could support the paper-chips. As the paper was stacked into the printer, multiple 7 mm-diameter paper chips were fabricated at the same time to align with the hole in the plastic support module. Thus, the fabricated PCD, which combined the plastic support module with the paper-chip, was able to be used for Hg^2+^ assay.

### Digital Images and Dates collection

We obtained the results of the PCD asaays with a digital camera and a fiber optic device. The camera was used to clearly capture the color information of the paper-chip. Moreover, we selected a fiber optic device as a protable and off-site tool which could transmite the color change to a digital value for Hg(II) analysis. Image of the fiber optic device with the PCD sensor was showed in Fig. S3, Support Information. For real-time result analysis, we vertically fixed a fiber optic probe above the detection zone of PCD at height of 0.3 mm. This made it suitable for accurately date acquisition. In principle, the probe emit the red laser, identify the reflective laser intensity and present it as a digital pattern simultaneously, thus light-colored paper-chips reflect most red laser and deep-colored chips would absorb more light which could induce a remarkable result discriminations.

### Colorimetric detection of Hg^2+^

For method development, several relatively simple procedures were followed. First, we used a plastic dropping bottle (5 ml) as a reactor to mix the samples containing Hg(II) with the PtNPs solution. As the Hg(II) was diluted in deionized water to specified concentrations, 2 ml of the Hg(II) sample was added to the dropping bottle to effect rapid reaction (2 min.) with the PtNPs solution (1 ml). One drop of reagent (ca. 10 μL) from the dropper bottle was dispensed onto the detection zone where 10 μl of TMB/H_2_O_2_ colorimetric substrate solution had already been placed. The paper underwent complete reaction within 5 minutes and the colored images were captured by digital camera or smartphone and where the specific color intensity of the paper was measured by the fiber optic device.

### Real sample analysis

Real samples of pond water and tap water were collected in 10 mL bottles and measured by the PCD (Support Information Fig. S3). The water samples were spiked to give a Hg(II) concentration of 0.025 uM and then mixed with PtNPs solution. After waiting 2 minutes, one drop of solution was placed on the detection zone of the paper chip and allowed to react completely. The colored papers were analyzed and all analyses were performed at least three times.

## Additional Information

**How to cite this article**: Chen, W. *et al.* A Simple Paper-Based Colorimetric Device for Rapid Mercury(II) Assay. *Sci. Rep.* **6**, 31948; doi: 10.1038/srep31948 (2016).

## Supplementary Material

Supplementary Information

## Figures and Tables

**Figure 1:**
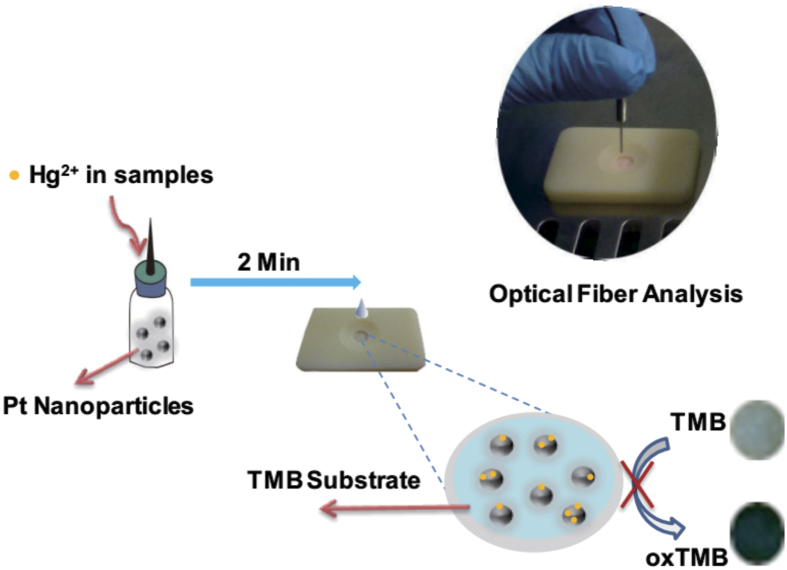
Schematic Representation of PCD for Rapid Hg^2+^ Assay.
