# Supplementary material for: A Simple Paper-Based Colorimetric Device for Rapid Mercury(II) Assay
Source: Sci Rep. 2016 Aug 24;6:31948. doi: 10.1038/srep31948 (PMC4995402; doi:10.1038/srep31948)
Supplement: Supplementary Information [file srep31948-s1.pdf]

Supporting Information

A Simple Paper-Based Colorimetric Device for Rapid Mercury (II) Assay

Weiwei Chen Xueen Fang*, Hua Li, Hongmei Cao, Jilie Kong*

Department of Chemistry and Institutes of Biomedical Sciences, Fudan University, Shanghai 200433, P.R.China.

* Correspondences should be addressed to J.L.K. (jlkong@fudan.edu.cn) and X.E.F. (fxech@fudan.edu.cn).

Supplementary Note 1

PCD assay was performed as reported in experimental section just with different reaction temperature or time. As seen from Figure S1(Up), the PCD method showed all good performances at four different reaction temperatures. And in figure S1(Down), we chosed five minutes as the reaction time because it was enough for complete oxidation of TMB at this PCD assay.

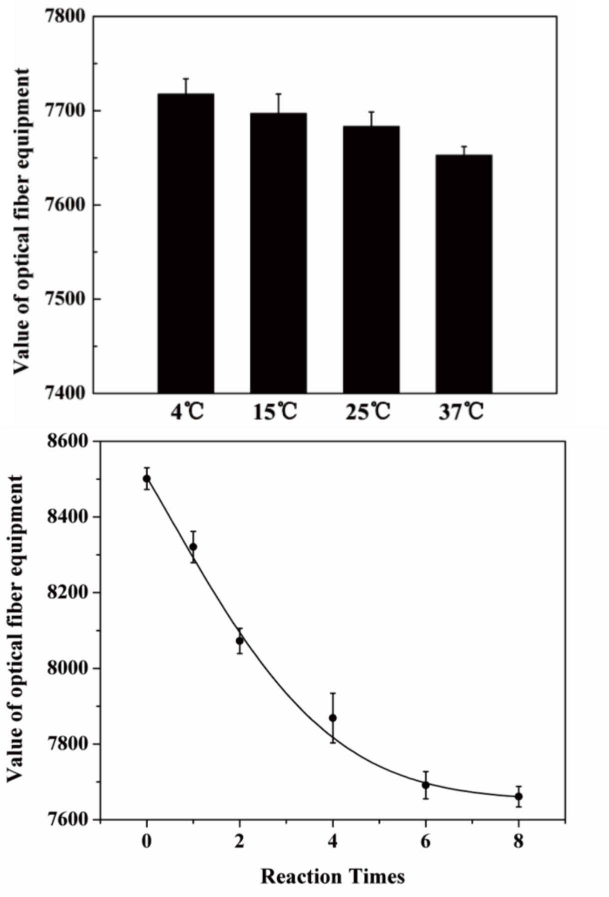

Figure S1: Effect of (Up) the temperature and (Down) the reaction time on the value response to the color intensity for PtNPs catalytic oxidation of TMB on PCD. Each point represents the mean value $\pm$sd (n = 3).

It can be seen from Figure S2 that two minutes is perfectly ensure the interaction between Hg^{2+} and PtNPs as its oxidation capacity was extensively suppressed.

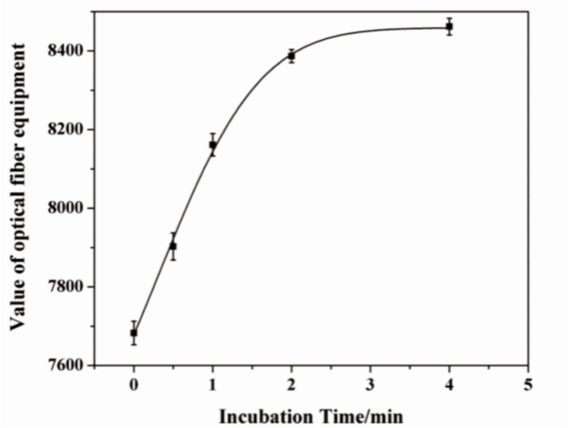

Figure S2: Optimization of incubation time. The value obtained from optical fiber equipment refers to the color intensity response of different incubation times. Each point represents the mean value ± sd (n = 3).

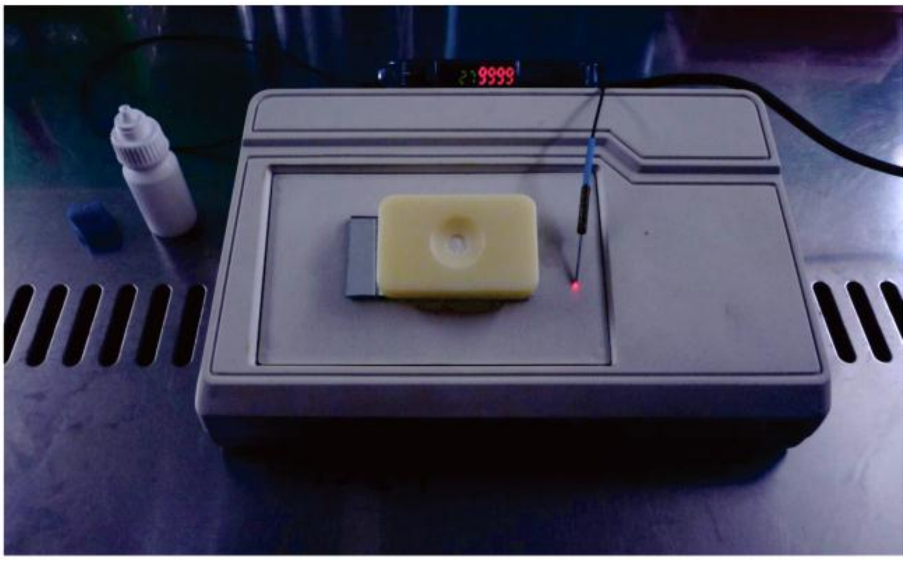

Figure S3: Photograph depicting the apparatus and method for real sample analysis by paper-based colorimetric device (PCD) performed for this work.

Supplementary Note 2

Table S1: Recovery experiments of Hg^{2+} in different water samples(pond water samples (1) and tap water samples (2) (n=3 for standard deviations)

Samples	Hg^{2+} added (uM)	Hg^{2+} detected (nM) by this PCD	Recovery (%)
1	0.025	0.0246 ±0.004	98.4 ±16
2	0.025	0.0220 ±0.003	88 ±12

Supplementary Note 3

As showed in Figure S4, the absorbance spectrum of PtNPs was general agree with other reports[1,2] that no obvious absorbance peak can be found. We also confirmed that peroxidase-like activity remained almost unchanged even the PtNPs were stored for up to six months.

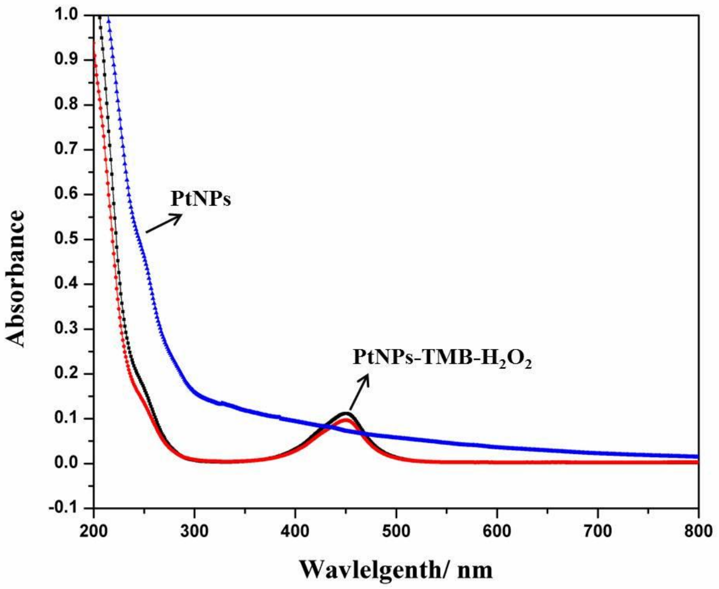

Figure S4 : UV-vis spectra of PtNPs and TMB oxidation catalyzed by PtNPs(fresh prepared) and PtNPs(six month later) in a PH 4.0 acetate buffer.

Reference:

1 Gao, Z., Xu, M., Hou, L., Chen, G. & Tang, D. Irregular-shaped platinum nanoparticles as peroxidase mimics for highly efficient colorimetric immunoassay. *Anal Chim Acta.* **776**, (2013).

2 Wang, G.-L., Shu, J.-X., Dong, Y.-M., Wu, X.-M. & Li, Z.-J. An ultrasensitive and universal photoelectrochemical immunoassay based on enzyme mimetics enhanced signal amplification. *Biosens. Bioelectron.* **66**, (2015).
